# Optimization of a Dexamethasone and Vitamin E PLGA Microparticle Formulation Intended for Posterior Segment of the Eye to Cope with Patients’ Anti-Inflammatory Needs

**DOI:** 10.3390/pharmaceutics18101239

**Published:** 2026-09-29

**Authors:** Javier Rodríguez Villanueva, Rocío Herrero-Vanrell, Irene Bravo-Osuna, Marta Vicario-de-la-Torre, Manuel Guzmán Navarro

**Affiliations:** 1 Unidad Docente de Farmacia y Tecnología Farmacéutica, Departamento de Ciencias Biomédicas, Facultad de Farmacia, Universidad de Alcalá, Ctra. de Madrid-Barcelona A-2, Km. 33,600, 28871 Alcalá de Henares, Spain; 2 Departamento de Farmacia Galénica y Tecnología Alimentaria, Universidad Complutense de Madrid, Plaza de Ramón y Cajal, 28040 Madrid, Spain

**Keywords:** microspheres, biodegradable, intravitreal administration, dexamethasone, small-gauge needles, controlled release, anti-inflammatory, personalized medicine

## Abstract

**Background**: Biodegradable microspheres (MSs) are good candidates for intravitreal administration in the posterior segment of the eye. MSs can be formulated as injectable preparations and administered using syringes with small-gauge needles. To allow proper injectability and avoid patients’ discomfort, the MS size should be as small as possible. However, the relationships between size, encapsulation efficiency, and drug-release behavior in these systems are widely recognized. **Methods**: In an attempt to optimize this requirement, we present here interesting dexamethasone (Dxm), vitamin E and human serum albumin (HSA) biodegradable MSs in the 2–20 µm range, elaborated by employing a version of the single-emulsion extraction–evaporation method. The method employed here renders MSs in the 2–40 µm range. The present work focuses on evaluating the smaller fraction, the 2–20 µm range, including physicochemical characterization, image analysis, dexamethasone release assays, cytotoxicity, and injectability evaluations. **Results**: The 2–20 µm fraction represents 25–30% of the entire population, has an average particle size of 18 µm, is spherical in shape, and has a Dxm loading between 38 and 50 µm per mg of MSs. In addition, a comparative evaluation between both fractions was carried out to assess whether relevant therapeutic differences can be expected. The in vitro release studies showed that MSs sized 2–20 µm, when vitamin E and HSA were included, have a low burst effect and, after that, a biphasic release profile during the first 4 weeks, with a long sustained Dxm release phase. This suggests that they might be better suited to maintain therapeutically anti-inflammatory concentrations of Dxm in the vitreous than the 20–40 µm MSs fraction, which also had a controlled Dxm release but in very reduced amounts after the first week. **Conclusions**: This 2–20 µm MS system might represent a success for its advantages over the existing alternatives in the marketplace, such as dexamethasone implants. Among others, due to the minimized risk associated with their administration that does not need ocular surgery, and consequently, the better the patient’s adherence is expected, there can be a medical preference for this system for its ease of use and possibility of personalizing the dose.

## 1. Introduction

In ocular pharmacology, one of the biggest challenges is achieving adequate sustained drug release over extended periods [1,2,3]. This is particularly important in the treatment of pathologies that affect the posterior segment of the eye, where drug access is limited [4,5]. Among them, inflammatory and immune-mediated ocular diseases represent an important cause of visual morbidity and may lead to significant visual impairment and blindness [6].

To address these conditions, Poly (lactic-co-glycolic acid) (PLGA) is the most commonly used polymer in clinics due to its excellent biocompatibility and biodegradability, and Dexamethasone (DXM) is a potent type of synthetic glucocorticoid corticosteroid widely used in ophthalmology [7]. Novel strategies involving PLGA and DXM include hydrogel approaches [8,9], microneedles [3], intraocular lenses [10] or micelle-gel systems [11]. Also, nanoparticles [12] or implants have been designed [13]. The most clinically important example is Ozurdex^®^, in which DXM is embedded in a 50:50 PLGA matrix. Those tools’ main difficulty is to obtain predictable, sufficiently sustained and reproducible release profiles [14].

Microspheres (MSs) constitute an important part of particulate drug-delivery systems that have demonstrated their potential for treating a variety of ocular conditions [2,15,16,17]. They can be formulated as injectable preparations and administered using syringes with small-gauge needles [18]. Among the advantages of MSs, it is worth highlighting that they are able to maintain effective active-substance levels over long periods [19,20,21]. Furthermore, these systems are good candidates for personalized medicine, as they allow each patient to be administered the appropriate amount of drug by adjusting the concentration of MSs [22].

The encapsulation of therapeutic proteins and peptides in microparticles has emerged as a promising strategy for the treatment of ocular diseases [23]. Double emulsion is the primary method for encapsulating hydrophilic proteins within hydrophobic PLGA [24]. W/O/W double emulsion solvent evaporation is the principal approach for hydrophilic proteins such as bovine serum albumin or LRP-based therapeutics [25,26]. The critical advantage is that the protein remains in an aqueous compartment while the PLGA occupies the organic phase. This feature reduces the problem caused by organic solvent exposure in protein structure and activity. On the other hand, drug inactivation, low drug loading, burst effect, and insufficiently sustained release account for the main drawbacks of protein microencapsulation [27].

Drug-release kinetics on multiparticulate drug-delivery systems are governed by a large number of factors related to the particles, such as size, surface area, porosity, morphology and internal structure (e.g., drug distribution and drug−matrix interactions), as well as to the nature and characteristics of the release medium (e.g., pH and ionic strength) [25,28,29]. In particular, diffusion and erosion mechanisms control the release of drugs from microparticulate delivery systems prepared with PLGA [30,31]. Some years ago, Li and colleagues [32] demonstrated that PLGA degradation was the dominant factor in governing drug-release kinetics from MSs. More recently, Gasmi et al. [33] explained the observed triphasic drug-release patterns for MSs of the same size that were loaded with different drug amounts: an initial burst release followed by a lag period and then a second burst release. The first burst was related to the dissolution and diffusion of the drug at or near the surface of the microparticles; the lag period was related less to morphological changes than to physicochemical changes in the polymer, which contributed to the increasing mobility of the drug molecules. The second burst was related to significant erosion of the polymer matrix and to an important change in the trend of porosity, thus indicating that porosity plays a crucial role in controlling drug release.

When temperatures exceed 37 °C, PLGA degradation proceeds homogeneously. However, the ocular temperature of healthy eyes typically ranges from 31 to 37 °C [34], and degradation rate and porosity depend on the polymer, on the process used to obtain the particles and, notably, on the system size [25,35]. Due to PLGA’s high load-encapsulation efficiency and prolonged release time, relatively large MSs are preferred for intravitreal injection [18,36]. Nevertheless, intravitreal injection requires small-gauge needles to minimize injury [37]. For all these reasons, an accurate understanding of the relationship between size and release profile is essential in the creation of MSs that are useful and of an appropriate size for therapeutic applications [38].

To improve the controlled release of Dxm, a new generation of biodegradable PLGA MSs, loaded with vitamin E and human serum albumin (HSA), has been developed [39,40]. It has been shown that serum albumin and vitamin E, when incorporated in solid state, reduce the initial burst effect and release rate of drugs [40]. Oily substances, such as vitamin E, have been demonstrated to not only increase encapsulation efficiency but also prolong drug release time [41]. In addition, vitamin E is a natural antioxidant with anti-proliferative activity and regulatory effects on protein kinase C, a key point of glutamate transport of great interest in ocular neurodegeneration, and might also exert synergic pharmacological effects [42]. Serum albumin, for its part, contributes to maintaining the integrity of the encapsulated drug, exerting a stabilizing effect during preparation and conservation [43].

MSs were prepared according to an S/O/W encapsulation method [44]. A modified version of the emulsion solvent extraction/evaporation method was used. In the first phase of the research, the particles with sizes between 20 and 40 µm were selected based on the mean particle size, the encapsulation efficiency, and the DXM release profile to develop an extended-release formulation for administration in the posterior segment of the eye. They demonstrated that this might be a useful tool to avoid the progression of degenerative ocular diseases [39,45]. However, the preparation method also renders a population of particles ranging from 2 to 20 µm, which could be very interesting from the biopharmaceutical point of view (due to these particles’ release rate) and from the pharmacotechnical point of view (due to their potential injectability characteristics, which offer possible benefits in clinical practice). The present work focused on evaluating the smaller 2 to 20 µm fraction, including physicochemical characterization, image analysis, dexamethasone release assays, cytotoxicity and injectability evaluations to assess whether relevant therapeutic differences can be expected between fractions when administered in vivo.

## 2. Results

### 2.1. Microsphere Characterization

Three MS formulations loaded with Dxm were prepared with the components and in the quantities listed in Table 1; for each formulation, two fractions (2–20 μm and 20–40 μm) were collected. All studies carried out in this research were performed with MSs in the 2–20 μm range, while MSs in the 20–40 μm range were characterized elsewhere [44].

When emulsifying conditions were set as described (5000 rpm for 1 min), the microencapsulation method caused the MSs to form a unimodal distribution of particle size, and the microencapsulation process had an overall yield higher than 90% for all formulations (Table 2).

The mean particle size for the three formulations showed no statistically significant differences (*p* > 0.01), with the average size (mean size ± standard deviation) being 18.1 ± 0.5 μm (Table 2). Optical microscopy observation of the MSs suspensions revealed an absence of Dxm crystals in the medium, indicating that all the drug had been encapsulated. SEM showed, for all three formulations, spherical particles with smooth surfaces (Figure 1).

When vitamin E was included in the inner-O phase (F2 and F3), some small pockmarks were observed on the MSs’ surfaces. To identify protein distribution, fluorescently labeled human albumin (FITC-HSA) was chosen for confocal studies. Figure 1 shows that FITC-HSA is located near the sphere’s surface in the 2–20 μm fraction. For the surface charge of the particles, a negative ζ potential was obtained for all three formulations (F1, F2 and F3). Table 2 summarizes these results, as well as the loading data for all prepared MS formulations. MS size was proportional to the drug loading. A high Dxm-encapsulation efficiency (more than 90%) and an encapsulation ratio close to 40 μg Dxm/mg MSs was achieved for all three formulations. However, this loading was partially affected by the inclusion of HSA. Consequently, the F1 and F2 MSs showed similar drug loadings (49.9 ± 4 and 47 ± 5.2 μg Dxm/mg MS, respectively), but both were statistically higher (*p* < 0.01) than F3, whose load was reduced to 38 ± 3.8 μg Dxm/mg MSs.

PLGA (MW 53,000 g/mol) showed a glass transition (Tg) temperature of around 52 °C. For pure Dxm, the observed glass transition was attributed to the amorphous fraction, whereas the melting endotherm was associated with the crystalline fraction. Pure Dxm showed an endothermic peak at 262.3 °C, corresponding to its melting point and indicating its crystalline nature; this reflects the values reported in the literature [46]. For HSA, there were no melting effects in the DSC curves. The thermogram of the physical mixture was similar to that of pure Dxm and the polymer, showing the two thermal events mentioned above, which is consistent with the expected Tg range reported for mixtures of Resomer^®^ 503 and DXM [47]. When Dxm was encapsulated in the MSs (F1, F2 and F3), the drugs’ melting points were significantly lower (around 215 °C) compared to those of raw materials and/or physical mixtures; the enthalpy of fusion was also reduced (Figure 2). The degree of encapsulated DXM crystallinity was calculated from the melting enthalpy of pure DXM (149.4 J/g) and that of the microparticles (3.5 J/g), taking into account the DXM loading. The estimated relative degree of crystallinity in the MS is 46.9%.

### 2.2. Microsphere Behavior in Release Media

The MSs were incubated in 2 mL of PBS with 0.1% sodium hyaluronate (pH = 7.4) release medium. This combination formed a fluid solution with a gel-like consistency similar to the vitreous humor. It was found to have a dynamic viscosity of 0.4 Pa·s, in the range of the average human vitreous viscosity of 0.5 Pa·s [48]. The MS dispersion remained stable for more than one minute without significant sedimentation. According to the technical details of the API manufacturer, Dxm solubility in the release medium (PBS, pH = 7.4) is approximately 10 mg/mL. Low percentages of sodium hyaluronate have been stated not to modify Dxm solubility [49].

Using centrifugation, MSs were recovered for evaluation at the established sampling times. Figure 3 shows the release profiles of MSs F1, F2 and F3, together with SEM images of their appearance over time. MSs retained their spherical, smooth surfaces for up to 2 weeks of incubation. Later, the particles began to aggregate, and morphological changes were observed, including the loss of the spherical forms and the fusion of some particles to form a lattice of deformed MSs. Compared to the other particles, the F3 MSs, after 28 days of in vitro assay, showed noticeably less degradation and had fewer pores on the surface.

MSs F1, F2 and F3 showed similar release behaviors, with a reduced initial burst effect followed by a biphasic drug-delivery profile. After 4 weeks of incubation, F1 and F2 MSs released almost 100% of the drug: 96.31 ± 2.76% and 88.96 ± 4.32%, respectively. F3 MSs showed significant differences (*p* < 0.01) relative to both F1 and F2 with regard to the burst effect (4 h) and the percentage released after 28 days of incubation at 37 °C in simulated ocular fluid, which was only 55.80 ± 1.31% of the Dxm content (Table 3).

### 2.3. Release Kinetics Analysis

To identify the kinetic model that most accurately described the dexamethasone release from the MSs, the experimental data were analyzed using several mathematical models, including conventional approaches such as the Higuchi and Peppas models, as well as more recently developed models, namely the Klose–Siepmann and Gallagher–Corringam models (Supplementary Material Figure S1). Although the conventional models yielded high correlation coefficients (r^2^ > 0.9), deviations from the experimental data were observed at the initial stage of the release profile. Given the limited burst release, the experimental data were subsequently fitted to a biexponential equation based on a biphasic release profile involving two concurrent mechanisms, namely diffusion and polymer erosion. The mean Dxm release rate (ΔQ_Dxm/Δt) was calculated over the time interval considered and expressed as µg Dxm/day. The parameters K1 and K2 corresponded to the first-order rate constants associated with each release mechanism, whereas a and b represented their respective coefficients. The complete biexponential equation is given below:

(1) $$\frac{∆\mathrm{Qdxm}}{∆t}=a\cdot e^{-K_{1}\cdot t}+b\cdot e^{-K_{2}\cdot t}$$

The value of the release rate constant K_1_ was significantly lower (*p* < 0.01) for F3 MSs than for the other two MS formulations (F1 and F2). For K_2_, no statistically significant differences between formulations were exhibited (Table 3).

### 2.4. Cytotoxicity Assays

In vitro cytotoxicity results obtained by the MTT technique were corroborated by flow cytometry, showing no statistical differences independent of the technique employed. Cell viability after the addition of MSs sized 2–20 µm is shown in Figure 4. Viability of the culture macrophages normalized to control surpassed 90% in all cases. No statistical differences (*p* > 0.05) were observed within the control (100% viability) when macrophages were incubated with Dxm solution (10 μM) or MSs (blank, F1, F2 and F3). It is a major achievement that the cytotoxicity assay results showed low levels of cytotoxicity in HeLa cells, and almost none when HSA was included in the formulation (99.11 ± 0.41% for F3 MSs, similar to 99.16 ± 0.18% for 10 μM Dxm).

### 2.5. Injectability

All of these MS formulations showed an appropriate injectability (≤12 N over 10 s) through 30 G and 32 G needles when dispersed in isotonic PBS or hyaluronic acid (0.1 and 1% *w*/*w*).

## 3. Discussion

In previous works [36,39,44], we developed a method for preparing PLGA MSs by a solvent extraction/evaporation technique, in which drugs and/or proteins are incorporated in a solid state in their initial stages, allowing efficient incorporation into the particles and low denaturalization of biological products. Dexamethasone is used here, dispersing first in dichloromethane before emulsification. The inclusion of vitamin E and HSA as technological adjuvants is a strategy to modulate Dxm release. Vitamin E is an oily substance that, when included as an additive in the internal phase, could hinder the release of the drug, affecting its migration through the MS. Furthermore, it is well-known that tocopherols play a suppressant role on protein kinase C, which regulates glutamate transporter activity [50,51]. The Age-Related Eye Disease Study 2 (AREDS 2) has shown that antioxidants, such as vitamins C and E, lutein, zeaxanthine, and zinc, reduce the risk of developing advanced AMD by 27% at the 10-year follow-up (https://www.nei.nih.gov/areds2 (accessed on 14 August 2026) and [52]). For its part, HSA has a great emulsifying capacity. In addition, when entrapped in a solid state, it can efficiently reduce burst effects and/or drug release and increase encapsulation efficiency through interactions and complexes with drugs. The microencapsulation polymer PLGA was selected in a ratio of 50:50 because this ratio was the most biodegradable [53,54].

When MSs are intended for the posterior segment of the eye, they must respect an upper limit. No MSs larger than 75 μm should be selected for intravitreal injection [55]. Small MS systems can be administered through small-caliber Gauge needles (28 G = 0.36 mm nominal outer diameter or lower), thereby decreasing intervention risks and patient discomfort [4]. By adjusting the key parameters of our preparation technique (emulsifying speed and time or maturation time), the high production of F1 MSs in a unimodal distribution with a large population in the 2–40 μm range became feasible (Table 4). PVA 1% w/v concentration in the internal water phase enhanced primary emulsion stability and protein encapsulation [56] and has not been modified in any of our assays.

These MSs were separated by filtration into two fractions, sizes 20–40 µm and 2–20 µm. Taking into account the relevance of particle size, a comprehensive study of the obtained 2–20 μm MS fraction was assessed in this work to evaluate if there was an improvement in MS properties or in the in vitro release behavior to make it a better candidate than larger-sized MSs for further in vivo studies.

Regarding MS characterization, no differences in morphology or mean particle size were observed between formulations F1, F2 and F3 (Table 2). SEM showed, for the three formulations, spherical particles with a smooth surface with some small pockmarks when vitamin E was included in the inner-O phase (F2 and F3). For its part, confocal studies showed differences in albumin distribution in F3 MSs sized 2–20 μm and 20–40 μm (Figure 1). FITC-HSA is located near the sphere’s surface in the lower-sized MSs and is homogeneously distributed throughout the entire matrix in larger MSs. According to Checa-Casalengua et al. [36], these changes are related to the MS’s formation mechanism. It is suggested that when a homogeneous O-phase droplet is introduced into the aqueous medium, the organic solvent rapidly diffuses into the surrounding water, leading to the formation of a hardened outer shell that confines the protein and, when present, oily additives within a concentrated region near the microsphere surface. The rate of this process is a key determinant of the resulting distribution. In smaller MSs, which exhibit a greater specific surface area, the organic phase is released more rapidly, thereby promoting the migration of proteins and oily additives toward and accumulation in the vicinity of the MS surface. Moreover, given that the gradient between the MS and the outer water phase is the driving force for HSA diffusion [57], at a high HSA loading level as obtained here, there must be more HSA distributed near the surface area of the MSs (Supplementary Material Figure S2).

The ζ potential influences MS’s surface properties and stability (aggregation phenomena). A negative range between –8 mV and –15 mV, as obtained for all formulations and sizes (Table 2), is required to avoid nonspecific interactions [58,59].

In Figure 5, the results of the yield and the amount of encapsulated drug between the 2–20 μm and 20–40 μm MS fractions are compared. As expected, drug loading for the 2–20 μm MSs fraction was lower than for the 20–40 μm MSs fraction for the same number of particles. Particle size is related to the energy applied during emulsification. An increase in stirring speed delivers greater energy to the system, resulting in a marked breakdown of the forming particle organic droplet, a small size of the MSs finally obtained, and lower encapsulation drug content and encapsulation efficiency, which are associated with a higher rate of solvent removal [60]. Decreased Dxm loading when HSA is incorporated into the 2–20 μm fraction may be explained by the same reasons described above concerning HSA distribution. Nonetheless, the encapsulation rate was higher than 90% in all formulations. The low solubility of Dxm in water (0.035 g/L, pH 7) and its partition coefficient (log P = 2.03 ± 0.6) [61,62] hindered drug diffusion through the external aqueous phase, as part of the maturation and consolidation of the MSs.

The physical state of Dxm in the MSs was evaluated by DSC analysis. Results indicate that a fraction of the Dxm content is incorporated in a noncrystalline, dispersed or amorphous state in the polymer matrix after partial solubilization of the drug in dichloromethane. A small reduction in the polymer glass transition (Tg) is observed for F3 MSs containing HSA in their composition (Figure 2). The high molecular weight of the protein may partially modify the PLGA polymer-chain conformation and liaising, resulting in decreased rigidity.

During the release studies, samples of the MSs were collected at different times and were observed by SEM, as shown in Figure 3. In Figure 6, a graphical comparison can be found between the MS formulations, their size, and the phases observed in the release process. In vitro release studies showed a high particle-size–release dependency after the second week. Thereafter, the amount of Dxm released each week (µg/mg MSs) showed no significant differences (*p* < 0.05) between F1 and F2, but it was reduced almost by half in F3. This is different for the fraction sized 20–40 µm, where the amount released after a week is similar in each formulation, except for the first seven days (less than 39 µg for F1 and F2, and almost 13 µm—a third—for F3) (Figure 7).

During the first 4 h (burst effect) when the aqueous medium has direct access to the MS’s surface, a drug boost is observed. F3 led to the lowest release, only 2.45 ± 1.47 µg Dxm/mg MSs. After that, a lag phase appears. Water penetrates the MS’s matrix, and pores begin to form through the polymer. In F1 and F2, it lasts around 5 days, while in F3, it is shorter, lasting no more than 3 days. The degradation of the polymer influences the last phase, right to the end, where the MSs swell and lose their spherical form. As can be observed in Figure 7B, the Q DXM released each day, regardless of the stage of the triphasic drug-release profile considered, is lower than the nominal DXM release rate of 7 µg/day reported for Ozurdex^®^ in clinical studies [63]. Note that the 2–20 µm F3 MSs showed better preservation of their spherical shapes and a reduced number of pores on the surface after 28 days of in vitro release assays, compared with F1 and F2 MSs. Human serum albumin has been related to a PLGA protective property by interacting with the polymer and its degradation products [64]. The protein could cause difficult water access to the ester linkage and, consequently, this reduces hydrolysis and polymer degradation.

Although the underlying mass transport mechanisms are complex and often not fully understood [65], drug release from microparticulate delivery systems prepared with PLGA is generally controlled by diffusion and/or erosion mechanisms [30,33,66,67]. The diffusion process is primarily determined by the morphology of the MSs and the distribution of the encapsulated drug, both of which are key factors governing the overall release profile. In contrast, microparticle erosion is influenced by several parameters, including the polymer characteristics, the particle production process, and the size of the system [31,68]. Accordingly, changes in MS size would not be expected to substantially affect the diffusion-controlled component of drug release, whereas particle size may have a more pronounced effect on the erosion-controlled phase. For PLGA, degradation occurs predominantly through hydrolysis of the ester bonds in the polymer backbone, a process that is autocatalyzed by the carboxylic end groups [69,70]. The degradation of the MSs initially involves a reduction in polymer molecular weight resulting from ester bond hydrolysis, leading to the formation of shorter polymer chains [71]. As degradation progresses and the polymer fragments become sufficiently small, the matrix develops greater porosity, allowing the resulting degradation products to diffuse out of the MSs and dissolve in the surrounding incubation medium. The effect of particle size on degradation can be explained by differences in the diffusion distance of the degradation products. In smaller MSs, the products generated within the polymer matrix can reach the particle surface more readily, facilitating their removal into the surrounding medium. Conversely, larger MSs present a longer diffusion pathway, which may promote the retention and accumulation of degradation products within the matrix and consequently influence the degradation process. [35,71]. Mass loss from the polymer material occurs, a highly porous structure comes up, and drug release is quickly provided. Fusion of the particles increased with incubation to form large particles with a smooth surface, which is the most thermodynamically stable conformation [72]. The fusion of MSs causes a higher exposure of the surface area of the systems and, in turn, degradation becomes easier [68,73]. For long-term assays, particle fusion takes place quickly, and a unique mass of polymer is formed.

MSs loaded with hydrophobic drugs such as Dxm—a corticosteroid widely used in clinical practice as the treatment of choice in diseases that involve chronic ocular inflammation [1,15,74]—exhibit the typical triphasic release profile. The profile includes an initial burst release phase (drug dissolution), a lag phase (diffusion through the polymeric barrier), and a final phase of polymer degradation, where microparticle swelling takes place and the hydrophilicity of the system increases over time. A shorter-chain PLGA containing more COOH and OH groups is more hydrophilic than a longer-chain PLGA, resulting in an “inside-out” erosion. This facilitates water imbibition, leading to accelerated drug release [33]. PLGA MS formulations for long-term hydrophobic drug delivery are usually associated with a long lag phase characterized by a nearly constant drug-release rate [75] related to a constant drug concentration gradient between the saturated drug solution within the system (poor solubility and limited water for drug dissolution inside the MPs) and the surrounding bulk fluid [33]. In the present study, there was no marked burst effect, although this is generally observed and of relative importance for the high-load MSs presented here; two phases can be clearly distinguished. MSs in the 20–40 µm range showed a lag phase (for approximately seven days) and a controlled release phase until the end of the study, while MSs in the 2–20 µm range showed a shorter lag phase (for approximately 2 days) and a secondary zero-order release phase (Figure 6).

Release data were fitted to classical kinetics models, (i) the simplified Higuchi model (Q_t_ = K·t^1/2^), where K is the Higuchi dissolution constant, or (ii) Peppas (Q_t_/Q_∞_ = K·t^n^), where K is the release rate constant and n is the release exponent that characterizes the release mechanism of the drug, and more modern ones, (iii) Klose–Siepmann, a model based on Fick’s second law of diffusion that considers the geometry of microparticles and the following initial and boundary conditions ∂c/∂t = ∂/∂x (D ∂c/∂x) + ∂/∂y (D ∂c/∂y) + ∂/∂z (D ∂c/∂z), where c and D are the concentration and the diffusion coefficient of the drug [76], and (iv) Gallagher–Corringam, a model that comprises the initial ‘burst effect’ of a drug non-bound to the drug matrix and following slow release determined by the matrix erosion f_t_ = f_t max_ (1 − e^−k1t^ +(f_t max_ − f_B_)(e^k2t − k2t2 max^/1 + e^k2t − k2t2 max^)), where f_b_ is the amount of released additive in the first stage (burst effect), k_1_ the first-order rate constant at the first stage, k_2_ the second stage rate constant, and t_2 max_ the time of maximum release rate at the second stage data [77,78].

The correlation coefficient r^2^ was chosen to define the approximation accuracy of each individual model. Acceptable correlation is achieved when r^2^ values are equal to 0.9 or higher. In all cases, correlation coefficients are between r^2^ = 0.88 and r^2^ =0.92. The best linearity in 2–20 µm fractions was found for drug remaining versus time and Hixson–Crowell model plots, W_0_^1/3^ − W_t_1^/3^ = κ t, where W_0_ is the initial amount of drug, W_t_ is the remaining amount of drug at time t, and κ is a constant incorporating the surface/volume relation (either r^2^ = 0.91), associated with morphology changes during drug release. In the 20–40 µm fractions, it was found for the drug remaining or drug released versus time plots drawn (either r^2^ = 0.91). For the Peppas–Sahlin model, R square values higher than 0.95 were obtained. The Peppas–Sahlin model accounts for both Fickian diffusion of Dxm from the MS surface and Case II relaxation associated with the PLGA matrix. For the second release phase, extending from day 2 to the end of the study for the 2–20 µm MSs and from day 7 onward for the 20–40 µm fraction, the release profiles were best described by zero-order kinetics across all formulations, in agreement with the findings reported by Gu et al. [79]. Linear relationships were obtained, with R^2^ values above 0.95. Zero-order kinetics are indicative of a concentration-independent release process, suggesting that the rate-limiting step is predominantly determined by the structural characteristics of the polymer matrix rather than by the concentration of Dxm within the system.

As previously proposed, two morphological phases should be distinguishable; the existence of two phases was undoubtedly confirmed by SEM studies (Figure 3). The first phase has no conformational change and diffusion governing; as for the second phase, erosion is predominant, and MSs lose their spherical form. To address both processes, a biexponential summation equation was proposed. K_1_ and K_2_ coefficients are, respectively, rate constants for diffusion and erosion. As previously stated, no significant changes in diffusion were expected to be influenced by MS size. However, lower K_1_ values for the F3 formulation were obtained. The slower release observed for F3 MSs is possibly due to the Dxm–HSA interaction in the MS matrix [44]. As expected, K_2_ values for F1 and F2 20–40 μm fractions were half of those for the 2–20 μm fractions (0.173 ± 0.025 and 0.183 ± 0.021, for F1 and F2 20–40 μm, respectively, in contrast with 0.351 ± 0.001 and 0.350 ± 0.001, for F1 and F2 2–20 μm).

A higher K_2_ was found in F3 MSs. In this formulation, HSA was incorporated in a solid state. As HSA dissolves, it leaves more pores and interconnecting channels, thereby facilitating the release of the remaining Dxm. For the 2–20 µm fraction, HSA situated near the surface is easily accessed by the dissolution medium, which fosters erosion. These findings are consistent with the reduction in the HSA extracted after the release assay (9.08 ± 0.85% and 5.47 ± 1.02% for the 2–20 µm and 20–40 µm fractions, respectively).

After 12 months of storage in lyophilized form at 4 ± 1 °C, no significant changes were observed in either particle size or drug loading. Likewise, the release profile evaluated after the 12-month storage period remained comparable to that of freshly prepared MSs, with the same two distinct release phases being observed. Moreover, the cumulative percentage of Dxm released at the end of the study did not differ significantly from that measured in the initial release assays (Table 3): 97.28 ± 1.93 from F1, 90.02 ± 3.17 from F2 and 44.71 ± 3.74 from F3. Nonetheless, the release rate was subtly higher in the first 7–10 days. Some authors correlate this finding with the first stage of the natural degradation going on in PLGA after 12 months by hydrolysis of its ester bonds through bulk or heterogeneous erosion due to residual water molecules [80].

Cytotoxicity assays per the MTT technique and flow cytometry on human macrophages and human epithelial carcinoma cell lines (HeLa cells) are common and widely recognized investigation techniques to ensure the absence of new design toxicity that interacts with biological systems [81]. These cells are, through apoptosis by predicted gene response, especially susceptible to extrinsic components and stress conditions, being a good tracer of compatibility and also, in the case of HeLa cells, of irritation or ocular tolerance [82]. Both cell types were used here to conduct a preliminary assessment of cytotoxicity prior to in vivo studies. In vitro cytotoxicity results are shown in Figure 4. The results were expressed as a percentage of cell growth with respect to the controls. J774 macrophages showed little sensibility to MSs, and no conclusion can be discriminated between formulations. Nonetheless, it must be noted that the cell viability of cultured J774 macrophages, normalized to the control, exceeded 90% for the techniques and formulations.

The negative effect produced by MSs in HeLa cell cultures was reduced for MSs containing HSA in their formulations. When HSA was incorporated, cell viability was not less than 95% (99.17 ± 2.01% for 2–20 μm fraction). The crucial role of HSA in the extracellular environment of cultured cells can be understood in terms of its antioxidant properties or the ability to promote cellular survival after apoptosis signals via a G couple PI3 K-dependent pathway [83], among others. This allows us to hypothesize that free HSA could increase cellular survival, even under adverse conditions, by interacting with the cell membrane.

A good MS’s formulation designed for the posterior segment of the eye must enable proper administration via a minimally invasive technique [84]. The theoretical dose of MSs required for administration in an animal model can be estimated from the relationship K_0_ = c_ss_ (0.693/t_1/2_) V_d_, where V_d_ is the vitreous distribution volume, t_1/2_ is the vitreous elimination half-life of the drug, and c_ss_ is the minimum Dxm concentration required for anti-inflammatory activity in the vitreous. Per these calculations, a K_0_ value of 0.15 μg dexamethasone/day was obtained for Sprague Dawley rats (V_d =_ 0.06 mL, t_1/2 =_ 6.3 h) to achieve the proposed C_ss_ of 1 μg/mL [74]. This is the minimum concentration of Dxm that the release from the MSs needs to sustain. Based on the in vitro release study and applying these calculations, it was estimated that 25 μg of F3, our most sustained release formulation, is able to deliver an adequate quantity of the drug through a single injection into the vitreous of an animal model, allowing therapeutic levels for at least 30 days (Figure 7). After a month, F3 MSs keep more than 40% of the Dxm load, and controlled release is expected to continue until degradation, as in F1 and F2 MSs. Based on the obtained Dxm release rate, therapeutic levels could be prolonged up to 1 month more, which allows separate time, even more, between intravitreal injections.

MSs in the 2–20 μm range enable the maintenance of therapeutic levels in the vitreous with a smaller amount of MSs than if MSs in the 20–40 μm range were administered. This provides several advantages. In addition to the high-gauge needle required in clinical practice and the consequent benefits for their administration [85,86], small microspheres are associated with no blurred vision or precipitation, kept suspended in the vitreous given easily predictable kinetics. Furthermore, patients need to be administered a smaller amount of MSs, which allows the retention of the possibility of an increased dose, personalizing medication according to their needs [62].

As 4 μL is the maximum recommended volume administered in the posterior segment of rat eyes, to achieve the dosage, 6.8 mg MSs/mL suspensions in different media were prepared for injectability tests. Andres-Guerrero et al. [19] reported that blank and loaded MSs tend to aggregate both in the Eppendorf^®^ tube and inside the syringe used to administer drug formulations, this feature being more pronounced in loaded MSs containing oily additives. MS injectability depends on the spheres’ properties, such as particle size or particle aggregation (ζ potential related), as well as the syringe, needle size, medium used for microparticle suspension, the use of a diluent, and concomitant properties [56]. Under the conditions described, MS formulations showed an appropriate injectability (≤12 N over 10 s) through 30 G and 32 G needles. Aggregation tendency increased over time, namely more than 30 min after suspension preparation. Thus, to ensure proper administration, the injection of MSs should be performed during the first 30 min after dispersion. This assay revealed that MSs are suitable for clinical use by intraocular administration without surgical incision, with minimal adverse effects on surrounding tissue.

## 4. Materials and Methods

### 4.1. Materials

PLGA 50:50 (Resomer RG503^®^, inherent viscosity: 0.32 dL/g) was provided by Boehringer Ingelheim Chemical Division (Ingelheim am Rhein, Germany). Dexamethasone (Dxm; 9-fluoro-11β,17,21-trihydroxy-16α-methylpregna-1,4-diene-3,20-dione) and vitamin E in viscous liquid form were purchased from Sigma-Aldrich (St. Louis, MO, USA). Fluorescein isothiocyanate-conjugated human serum albumin (FITC-HSA) was obtained from Tebu-bio (Barcelona, Spain), whereas polyvinyl alcohol (PVA; MW 72,000 Da) and sodium hyaluronate (solubility: 5.0 mg/mL; molecular weight: 1.8 MDa) were supplied by Merck KGaA (Darmstadt, Germany) and Aston Chemicals (Aylesbury, UK), respectively. Sterile well plates for cell culture were purchased from Cultex S.L.U. (Madrid, Spain) and used for the cell viability assays. J774 macrophages were maintained in RPMI 1640 medium supplemented with L-glutamine (2 mM), fetal bovine serum (10%), penicillin–streptomycin (2%), and gentamicin (0.4%), all obtained from Difco (Franklin Lakes, NJ, USA), together with L-arginine (1 mM) from Sigma-Aldrich (USA). HeLa cells were cultured in DMEM supplemented with fetal bovine serum (10%), penicillin–streptomycin (2%), and amphotericin B (2%), all purchased from Sigma-Aldrich (USA).

Methylene chloride (CH_2_Cl_2_), acetonitrile, and methanol, all analytical grade, were obtained from Scharlab (Barcelona, Spain) and used upon receipt. Distilled and deionized water (Milli-Q^®^, Millipore Corporation, Burlington, MA, USA) was used to prepare the solutions and buffers.

### 4.2. Methods

#### 4.2.1. Microsphere Preparation

MS preparation was performed using the solvent extraction–evaporation technique from a solid-in-oil-in-water (S/O/W) emulsion according to the procedure described previously [23] and the compositions listed in Table 1. To establish the most suitable conditions in which to prepare MSs, previous studies were performed in which various agitation rates, homogenization and maturation times were evaluated (Table 4).

Based on the previously described studies, the inner organic phase was prepared by dissolving 200 mg of polymer Resomer 503^®^ in 1 mL of CH_2_Cl_2_ using a vortex mixer. Subsequently, 20 mg of Dxm was incorporated into the polymer solution in all formulations, yielding the initial S/O dispersion. For formulation F2, 20 μL of vitamin E (as α-tocopherol acetate) was additionally introduced into the S/O dispersion. In the case of F3, 20 μg of FITC-HSA powder (for primary sequence, UniProt P02768) was first resuspended in 20 μL of vitamin E by sonication (UIS250v, Braun Labsonic, Melsungen, Germany) for 30 s in an ice bath to minimize the potential for protein alteration. The resulting organic phase was subsequently emulsified into an aqueous phase containing 2% PVA by homogenization at 5000 rpm for 1 min using a Polytron^®^ PT 3000 (Kinematica AG, Malters, Switzerland). MS maturation and consolidation were performed by adding the dispersed S/O/W system to 100 mL of PVA solution (0.1 *w*/*v*). The emulsion was continuously stirred for 3 h at room temperature until the organic solvent completely evaporated. Then, the MSs were vacuum-filtered, washed three times with water, frozen at –80 °C, and lyophilized (Cryodos^®^ Telstar, Barcelona, Spain). For further use, they were then stored at 4 ± 1 °C in airtight vessels containing silica gel. During these processes, the samples’ exposure to light was minimized to protect them from degradation. MS batches were prepared in triplicate. Blank MSs were prepared following the same procedure, except with the polymer PLGA. MSs were separated according to their particle size using filtration, under vacuum conditions, through three nylon filters with pore sizes of 2, 20 and 40 μm (Gilson Inc., Middleton, WI, USA) (Figure 8).

#### 4.2.2. Microsphere Characterization

The MS production yield of each fraction was calculated. Mean particle size (expressed as volume–density mean diameter) and particle size distribution were measured using light scattering in a Microtrac SRA^®^ (Microtrac Inc., York, PA, USA) following dispersion of the samples in an aliquot of Milli Q^®^ water. Particle size distribution is expressed employing Span factor [87]. Zeta potential (ζ) was measured following the dispersion of MSs in an isotonic NaCl solution with a Zetasizer Nano ZS^®^ (Malvern Inc., Malvern, UK). The external and internal morphology of the microparticles was analyzed using scanning electron microscopy (SEM) with a Zeiss DSM 950^®^ (Zeiss, Oberkochen, Germany). The scanning electron microscopy protocol followed is based on the one described by da Cruz and Coworkers [88]. Briefly, samples were fixed on standard SEM pin stubs, mounted with carbon glue, and coated with gold sputter under vacuum conditions in an argon atmosphere, all prior to observation at 20 kV. The MSs’ morphology was analyzed before and after the Dxm release assays. The finding that protein was encapsulated into the MS matrix was determined using confocal microscopy with a Nikon Eclipse TE300 MRC-1024^®^ (BioRad, Alcobendas, Spain). For that purpose, the samples were fixed with Prolong^®^ Gold resin (Invitrogen, Carlsbad, CA, USA) 24 h prior.

Differential scanning calorimetry (DSC) was carried out using a Mettler TC15^®^ (Mettler International, Cornellà de Llobregat, Spain) that had been previously calibrated with indium. The samples were accurately weighed (4–5 mg) and sealed into aluminum pans. Heating curves were recorded at a scan rate of 10 °C/min from 0 to 300 °C in a dry-nitrogen atmosphere. DSC thermograms of drug, polymer, physical mixture (10:1 ratio of Resomer RG503^®^ to Dxm), HSA, and microparticles (including the unloaded and F1-, F2-, and F3-loaded MSs of each fraction) were obtained.

#### 4.2.3. Loading Efficiency

The Dxm-loading efficiency of MSs was determined by dissolving 2 mg of freeze-dried MSs in 1 mL of CH_2_Cl_2_. The resulting solution was stirred for 4 h. Next, the polymer was precipitated with 96° ethanol (0.5 mL) and centrifuged (at 5000 rpm and 15 °C for 10 min). The supernatant was collected and filtered through a 0.45 µm cellulose acetate membrane (Sartorius 16555-K^®^, Barcelona, Spain). The procedure was repeated four times, and extractive liquids were collected. The drug was quantified using HPLC on the extracted solutions, as described below. The total amount of Dxm was calculated from the aliquots of each extract.

Drug loading was expressed as (weight of drug loaded/weight of microspheres) × 100%.

The HSA loading efficiency of F3 MSs was calculated using an independent procedure; 2 mg of freeze-dried MSs was dissolved in 0.7 mL of CH_2_CL_2_ through 4 h of mechanic stirring. Then, 1 mL of Milli-Q^®^ water was added and stirred again for 24 h, followed by centrifugation (at 5000 rpm and 15 °C for 10 min). The supernatant was removed, and another 1 mL of Milli-Q^®^ water was added. This process was repeated four times, with extractive liquids collected and freeze-dried in low-binding Eppendorf. The FITC-HSA content was redissolved in an aqueous buffer medium and quantified using spectrofluorimetry, as described below.

#### 4.2.4. Apparatus and Analytical Procedures for Dxm and FITC-HSA Quantification

##### Chromatographic Analyses

Chromatographic analyses were carried out using a validated method that was described in previous research [44]. The method is linear in the concentration range of 2.5–10 μg/mL, with its limits of detection and quantitation being 0.008 and 0.03 µg/mL, respectively.

Briefly, the solution was filtered (Millex^®^ HV, St. Louis, MO, USA, PVDF 0.45-μm syringe filter) and subjected to HPLC analysis with a 100 μL injection volume. A Beckman System Gold 508^®^ was equipped with two pumps, an autosampler, a UV/VIS detector, and Karat 32^®^ controller software (all from Beckman Coulter Inc., L’Hospitalet de Llobregat, Spain). The separation was achieved using a 3.9 × 150 mm W30391M094 C18 5 µm column (Waters Corp., Milford, CA, USA), which was thermostatically maintained at 45 °C using a Gecko 2000^®^ heating column (Amcro GmbH, Hattersheim am Main, Germany). Analysis was performed under gradient conditions with two mobile phases: acetonitrile and water in a 35:65 *v*/*v* mixture (phase A) and pure acetonitrile (phase B). Both were delivered at a flow rate of 1.0 mL/min. The initial mobile phase condition of 100% A and 0% B was progressively changed to 0% A and 100% B from *t* = 10 min to *t* = 25 min. Subsequently, the initial conditions were reinstated for the following 5 min, until the end of the assay (*t* = 35 min). The detection wavelength was set at 254 nm. None of the MS components interfered at this wavelength (Figure 9).

##### Spectrofluorimetric Method

Quantification of FITC-HSA in phosphate-buffered saline (PBS) was performed in a spectrofluorometer Shimadzu RF-540^®^ (Shimadzu, Kyoto, Japan). Conditions were set to 480 and 494 nm for the excitation and emission wavelengths, respectively. The calibration curve for FITC-HSA with standard solutions was established in a range from 0.05 to 0.2 μg/mL and with a limit of quantitation of 0.025 µg/mL.

#### 4.2.5. In Vitro Release Assays

An in vitro release study was performed under sink conditions. The methodology employed is based on the one described by Checa-Casalengua and coworkers [36]. MS samples (2 mg) were accurately weighed in low-binding Eppendorf^®^ tubes and filled with 2 mL of isotonic PBS—which had a pH of 7.4 and contained 0.1% sodium hyaluronate to simulate vitreous humor—0.02% Tween 80^®^ to facilitate particle dispersion, and 0.05% sodium azide as a preservative. The tubes were placed in a Clifton NE5^®^ water-shaker bath (Nickel-Electro Ltd., Weston-Super-Mare, UK) at 37 °C with a constant agitation of 60 strokes min^−1^. At scheduled time intervals (every 24 h for the first 3 days, and every 72 h after that), the tubes were centrifuged (at 5000 rpm and 20 °C for 10 min); the supernatant was then withdrawn with a syringe, and the Dxm concentration was determined by HPLC. The same volume of fresh medium was added to the tube to continue the release study. This release assay was performed in triplicate for each batch of MSs. The results were then fitted to various kinetic models.

#### 4.2.6. Cytotoxicity Assays

1. Methylthiazole tetrazolium method

The methylthiazole tetrazolium (MTT) assay, a quick, quantitative colorimetric test, was chosen to preliminarily screen the MSs for nontoxicity. This test reflects the metabolic activity of the cells that may not be directly related to cell death as an outcome. MTT was performed according to the procedures established in international guidelines and European directives (e.g., 200423/EC, 2004). Based on the preliminary results from an in vitro release assay, the test was performed in triplicate on 24 h cell cultures that had been incubated with 10 µM Dxm or with 20 µL of a 1 mg/mL PBS-particle suspension of each formulation and size. Microparticles without drug but with a solution of benzalkonium 0.005% were added under the same conditions and concentrations to control wells. J774 macrophages and HeLa cell lines were selected. J774 macrophages are a murine cell line, important mediators of inflammation and innate immune response with a strong phagocytic property. They can help determine whether a formulation induces macrophage toxicity or an excessive inflammatory response. HeLa cell lines were selected as a well-characterized and reproducible model for preliminary cytotoxicity screening. Their robust growth and established use in standardized cell viability assays facilitate the assessment of concentration-dependent cytotoxic effects.

Briefly, J774 macrophages and HeLa cell lines were cultured in 1 mL wells at 37 °C in a humidified atmosphere (95% air and 5% CO_2_) with a density of 1.5 × 10^4^ cells/mL. Each well was inoculated with 20 μL of the sample and then incubated for 24 h. After incubation, the medium was aspirated, and 100 μL of MTT solution (0.2 μg/μL in 0.2% dimethyl sulfoxide) was added to each well. After incubation in darkness at 37 °C for 1 h, the formazan crystals produced from the MTT were reduced to the mitochondrial cell level. The precipitated crystals, previously dissolved using 80 μL of a solution with 45% well dimethylformamide and 25% sodium dodecyl sulfate, were quantified via spectrophotometry. Absorbance values were measured by reading the plates at 570 nm on an ELISA plate reader (ASYS Hiteck GmbH, Seekirchen am Wallersee, Austria).

2. Flow cytometry

For an in-depth, more precise and reliable cytotoxicity evaluation, HeLa and MPH cells were seeded at 37 °C in 2 mL wells (within 6-well plates) with a density of 1.5 × 10^4^ cells/mL and then were incubated for 24 h. Next, 40 μL of the previously described samples were administered, followed by incubation for another 24 h at 37 °C. The cells were washed three times with PBS, detached using trypsinization, centrifuged, and then resuspended in PBS. Then, they were analyzed using flow cytometry. These measurements were performed using a FACSCalibur flow cytometer (Becton Dickinson, Franklin Lakes, NJ, USA).

#### 4.2.7. Injectability

To evaluate proper injectability, 6.8 mg of each MS formulation was suspended in (A) 1 mL of isotonic PBS with a pH of 7.4; (B) 1 mL of hyaluronic acid in isotonic PBS (0.1% *w*/*v*) with a pH of 7.4; and (C) 1 mL of hyaluronic acid in isotonic PBS (1% *w*/*v*) with a pH of 7.4. These tests were performed using a 1 mL syringe attached to 30 G and 32 G needles.

#### 4.2.8. Stability Study

Sufficient amounts of sample from all batches of MS formulations elaborated (F1, F2 and F3) have been stored for 12 months under controlled conditions: protected from light, in an airtight container including a moisture adsorbent of silica gel, and in cooling conditions at 4 ± 1 °C. Then, MSs were observed by microscope and analyzed in accordance with the procedures described in this section.

#### 4.2.9. Statistical Analysis

The statistical significance of the parameters (yield of production, loading efficiency, particle size, and cytotoxicity results) for each batch of MSs was tested using a one-way analysis of variance (ANOVA) with pairwise multiple-comparison procedures (Statgraphics Centurion XVII). Dxm-loaded MSs’ release profiles were compared by evaluating the similarity factor f_2_ (USP40-NF35, 2017). Differences were considered significant at a level of *p* < 0.05 and f_2_ < 90.

## 5. Conclusions

By employing a modified version of the solvent extraction–evaporation technique from a solid-in-oil-in-water (S/O/W) emulsion is feasible to obtain biodegradable MSs sized 2–20 µm loaded with Dxm, vitamin E and HSA not only with a good safety profile, biocompatibility by cell viability in HeLa cells and murine J-774.2 macrophages above 85% in all cases, but also with a low burst effect, only 2.45 ± 1.47 µg in F3, and a biphasic release profile in vitro over the course of 4 weeks. When HSA is incorporated in solid state, microparticles demonstrated an even slower controlled drug release, nearly one-third of the release rate from days 14 to 28, highlighting that HSA performs a modulating action with respect to Dxm release. The innovative strategy reported herein allows for the achievement of good MS candidates for further in vivo studies that may be useful systems for treating chronic degenerative diseases or inflammatory conditions in the posterior segment of the eye. For their small size, a small-gauge needle (20 to 30 G) can be used in clinical practice with consequent benefits for administration and fewer adverse effects (such as blurred vision or precipitation) associated with higher MSs. The MSs prepared here are demonstrated to be an adequate alternative to the existing alternatives in the marketplace, such as dexamethasone implants. They are expected to achieve better patient adherence and be a medical preference due to their ease of use and the possibility of personalizing the dose.

## Figures and Tables

**Figure 1:**
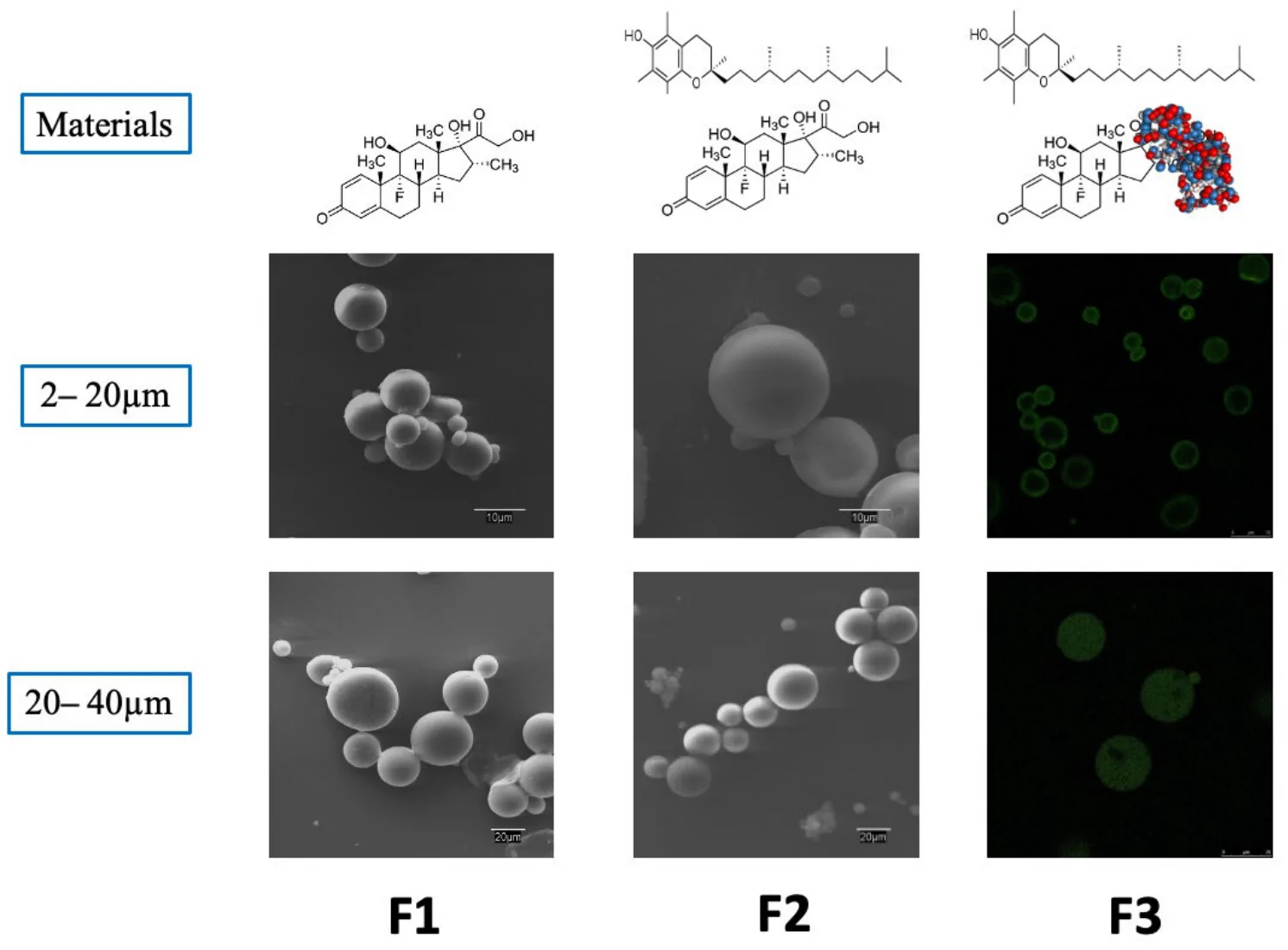
SEM images of F1 and F2 MSs fraction sized 2–20 µm (scale bar 10 µm) and 20–40 μm (scale bar 20 µm), and confocal laser microphotographs of F3 MSs fraction sized 2–20 µm and 20–40 μm. A schematic representation of each composition is included. Images from F3 MSs exhibit green fluorescence due to encapsulated FITC-HSA.

**Figure 2:**
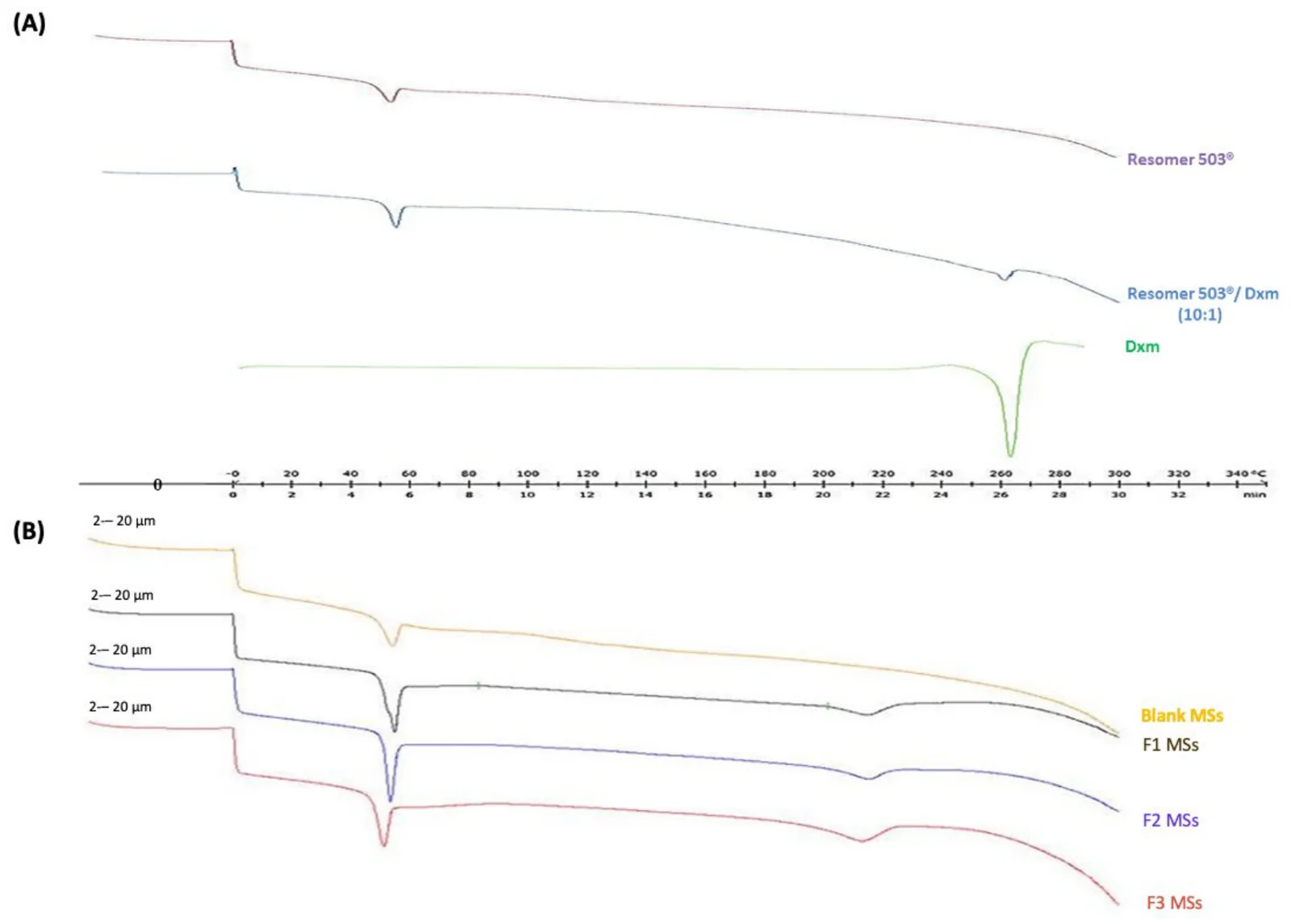
DSC thermograms (↑ Exo ↓ Endo, upward and downward direction of the event) recorded at a heating rate of 10 °C/min for (**A**) the raw materials, including PLGA (50:50), Dxm, and the PLGA/Dxm physical mixture (10:1), and (**B**) the blank MSs and Dxm-loaded MSs (2–20 µm) corresponding to formulations F1, F2, and F3.

**Figure 3:**
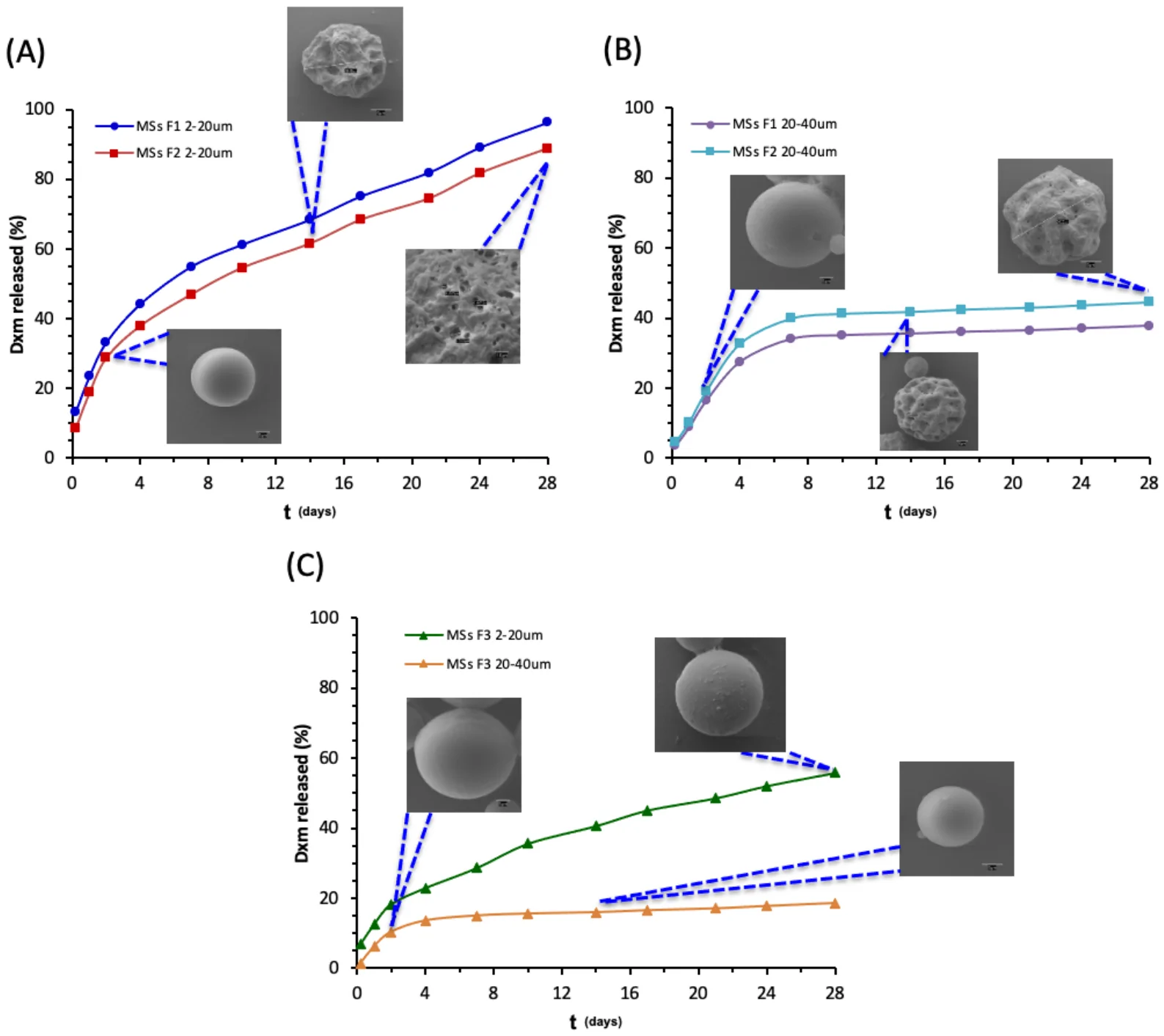
SEM images of 2–20 µm and 20–40 µm MSs during in vitro release assay. Morphology and surface after 48 h, in the middle of the study (Day 14), and after study conclusion (Day 28) are shown. (**A**–**C**) correspond to F1, F2 and F3 MSs, respectively.

**Figure 4:**
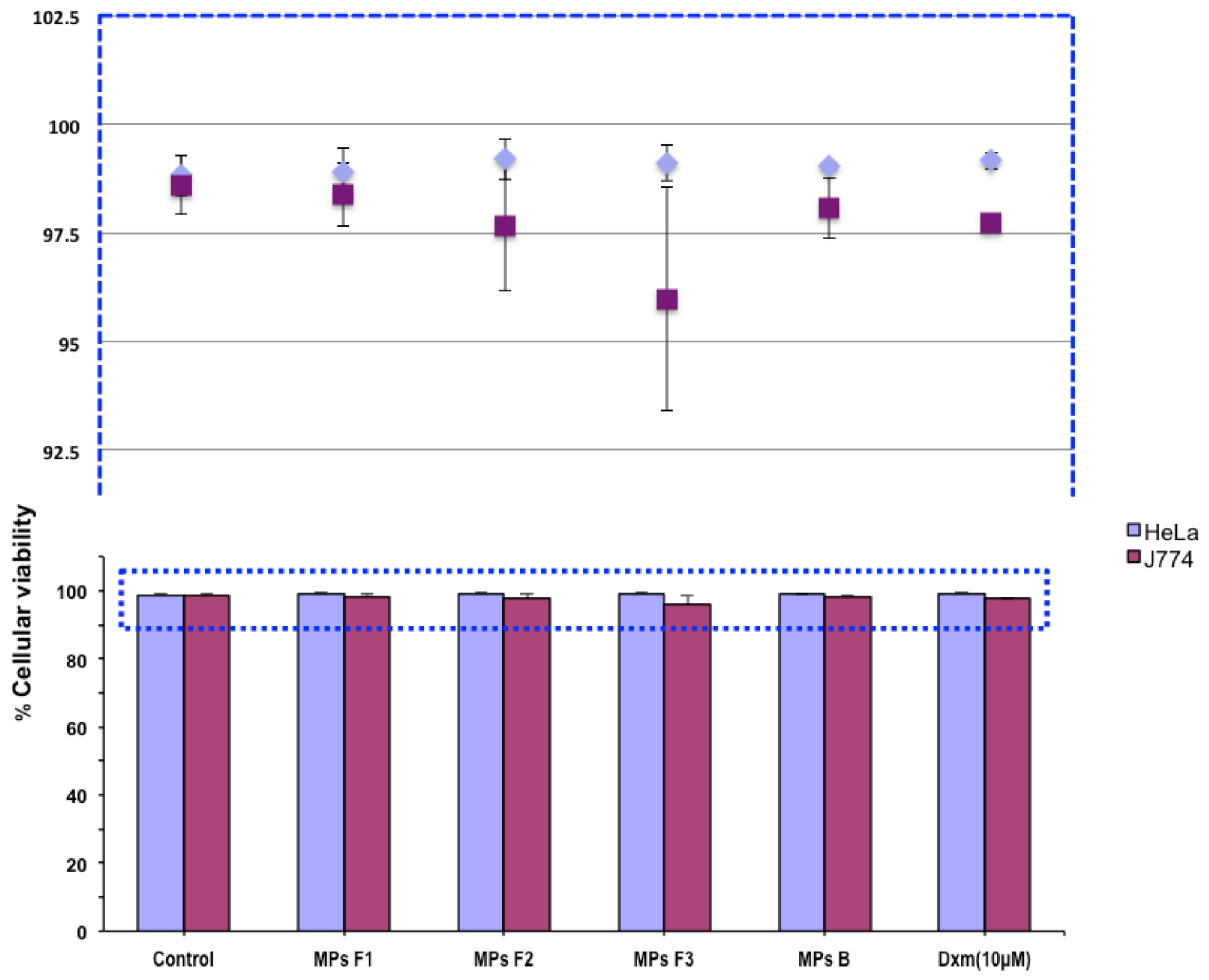
Cell viability (%) of J-774.2 macrophages (MPHs) and HeLa cells following treatment with free Dxm solution (10 μM), blank MSs, or Dxm-loaded MSs (formulations F1, F2, and F3), as assessed by flow cytometry. Pink bars correspond to MSs ranging from 2 to 20 μm, while purple bars represent the 20–40 μm MS fraction. * *p* < 0.05.

**Figure 5:**
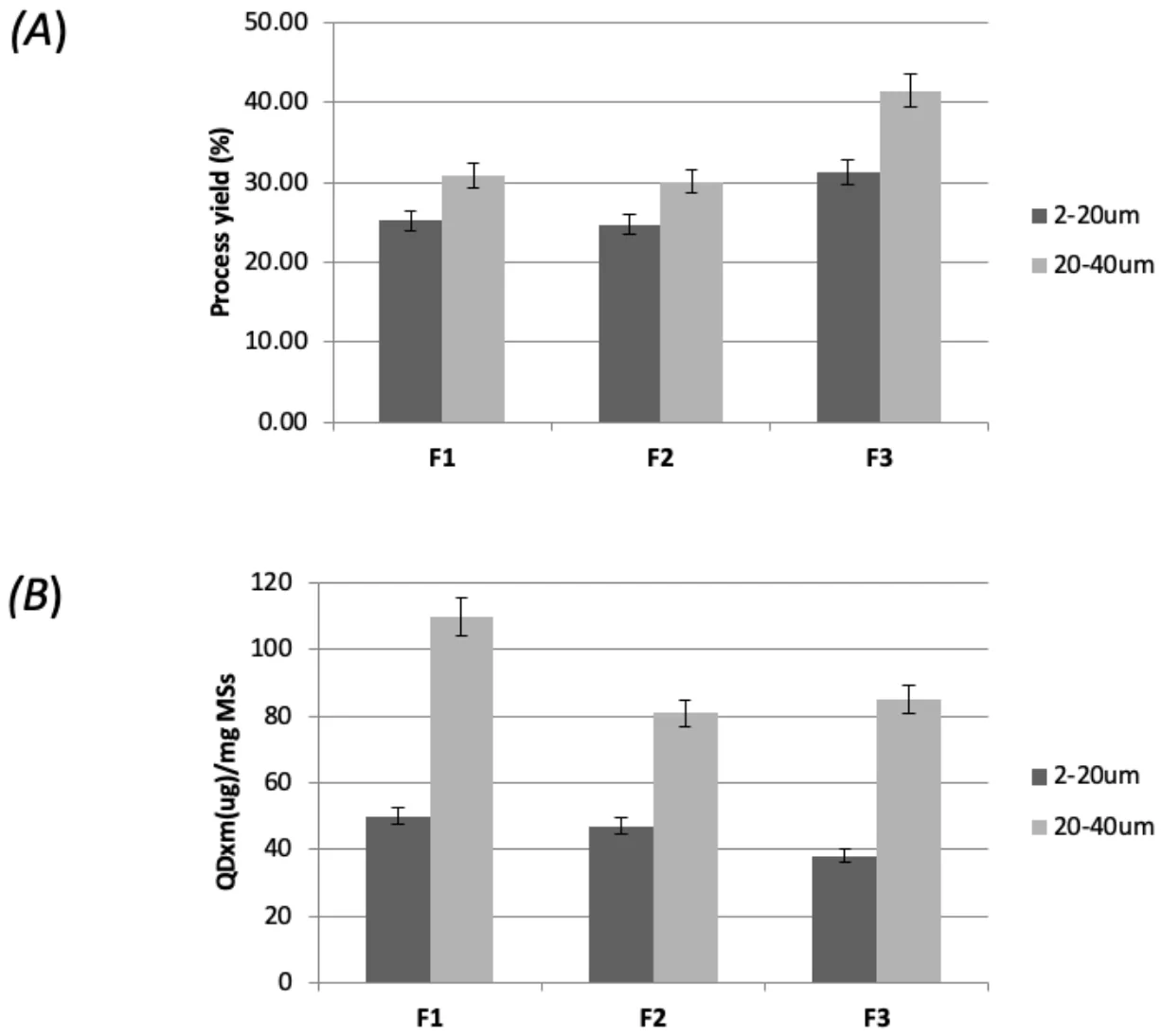
Comparison between (**A**) process yield (%) and (**B**) Q Dxm (µg/mg MSs) for F1, F2 and F3 2–20 µm and 20–40 µm fractions (data are shown as $\bar{X}\pm \mathrm{SD}$, n = 6).

**Figure 6:**
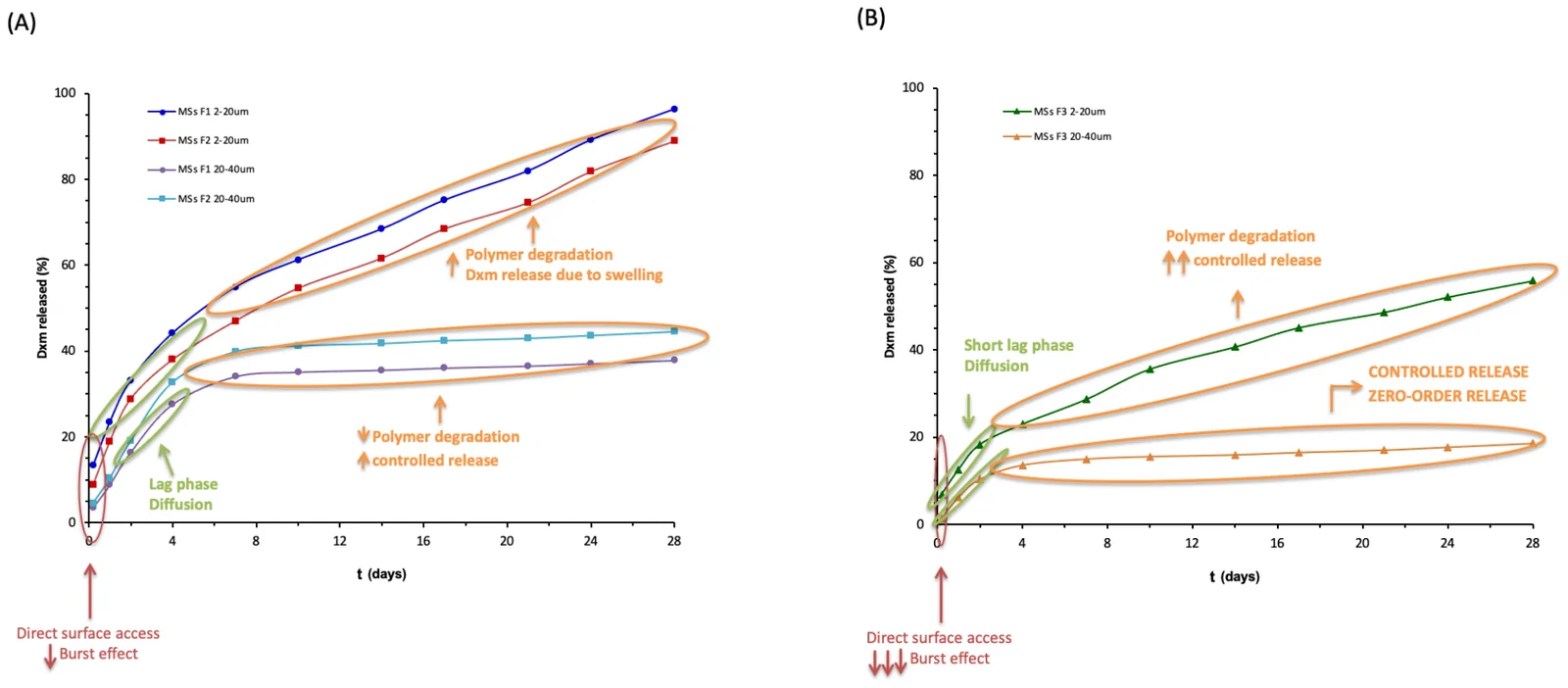
Analysis of the Dxm release profiles from MSs. (**A**) Comparison between MSs fractions sized 2–20 µm and 20–40 μm when only Dxm and vitamin E are included in the formulation. (**B**) Comparison between MSs fractions sized 2–20 µm and 20–40 μm including solid-state HSA in their formulation.

**Figure 7:**
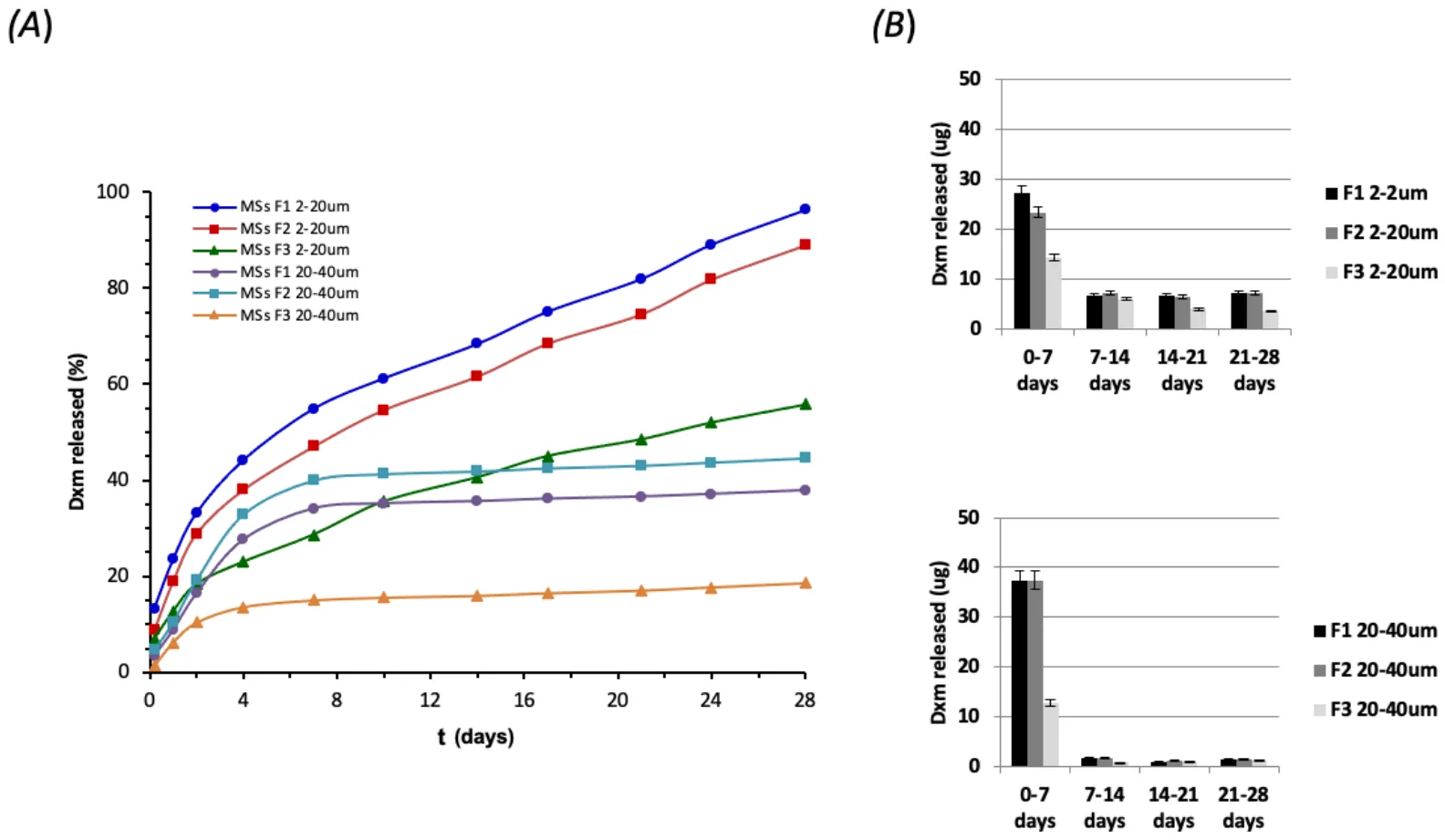
Q Dxm released each week (µg/mg MSs) during in vitro assays for F1, F2 and F3 2–20 µm and 20–40 µm fractions. (**A**) Dxm released (%) vs. t (days) (**B**) Dxm released (µg) vs. period (days).

**Figure 8:**
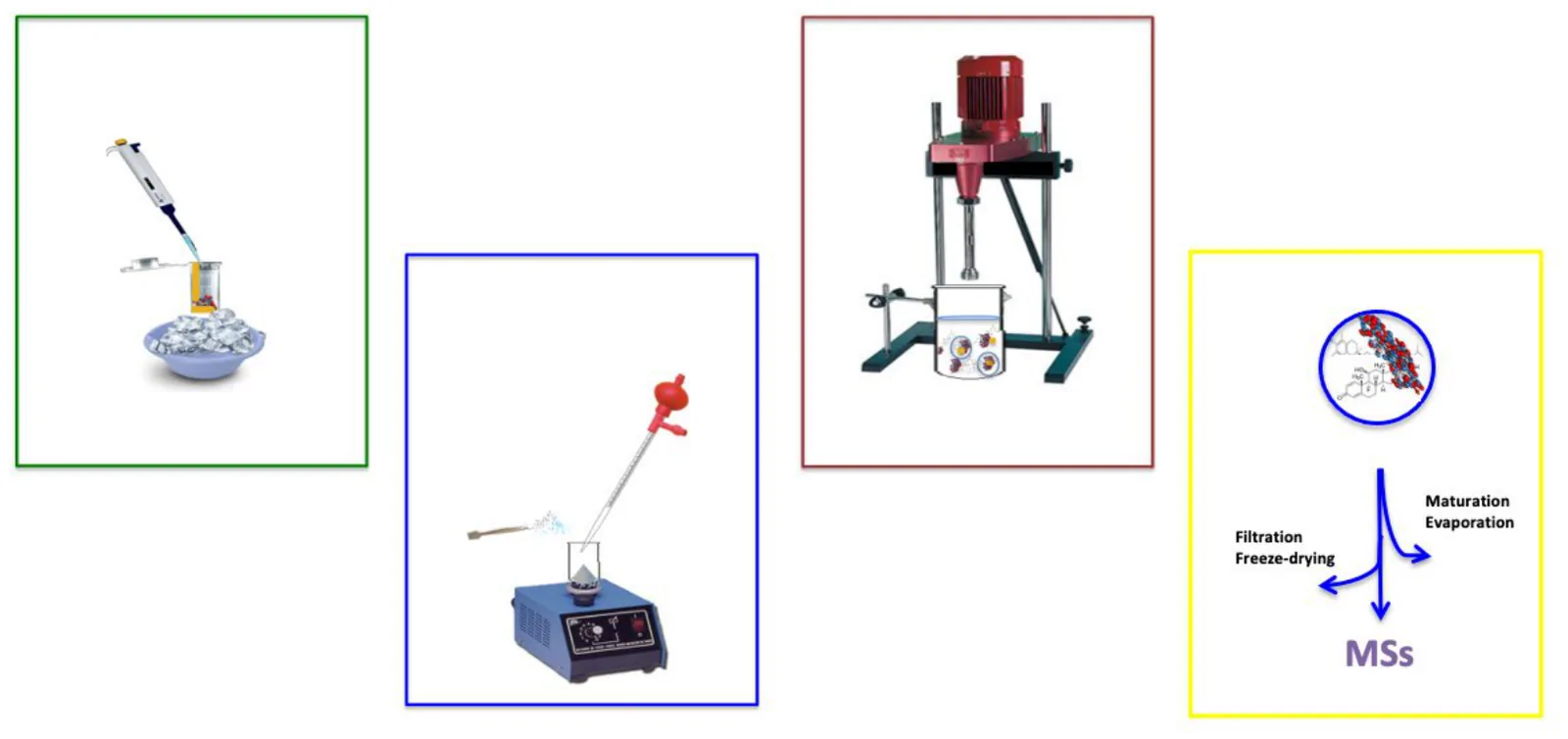
MS preparation performed using the solvent extraction–evaporation technique from a solid-in-oil-in-water (S/O/W) emulsion.

**Figure 9:**
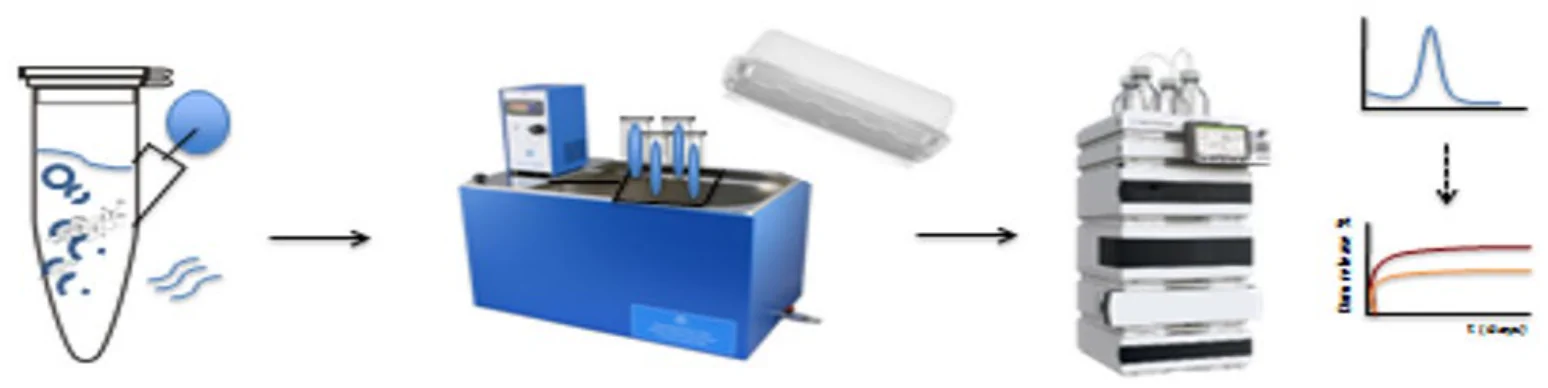
Apparatus and analytical procedure for Dxm quantification.

**Table 1:** PLGA (Mw 35,000 g/mol and inherent viscosity 0.32 dL/g, Resomer RG 503^®^) MS composition. Three Dxm MS formulations (F1, F2 and F3), plus a non-charged MSs (blank) as control, were developed.

MS Formulation	PLGA (mg)	Dxm (mg)	Vit. E (mg)	FITC-HSA (mg)
BLANK	200	-	-	-
F1	200	20	-	-
F2	200	20	0.02	-
F3	200	20	0.02	0.02

**Table 2:** Influence of MS’s composition on mean particle size, performance parameters of production and characterization of PLGA (50:50) 2–20 μm MSs, and batch-to-batch reproducibility of particle size and drug loading (data are shown as $\bar{X}\pm \mathrm{SD}$, n = 6) (^a^) Dxm (^b^) FITC-HSA.

Formulation	MSs Blank	F1	F2	F3
Mean particle size (μm) %CV	18.1 ± 0.3 4.8	17.9 ± 0.2 4.6	18.2 ± 0.3 5.1	18.0 ± 0.2 4.6
Production yield (%)	21.3 ± 4.5	25.0 ± 4.0	23.5 ± 2.1	30.7 ± 4.7
ζ-Pot NaCl 0.9% (mV)	−9.7 ± 1.0	−9.9 ± 1.0	−9.3 ± 0.6	−10.1 ± 1.0
Loading (μg/mgMS) Dxm ^(a)^ FITC-HSA ^(b)^	------	49.9 ± 4.3 ^(a)^	47.0 ± 5.2 ^(a)^	38.0 ± 3.8 ^(a)^ 0.070 ± 0.001 ^(b)^
Encapsulation Efficiency (%) Dxm ^(a)^ FITC-HSA ^(b)^ %CV	------	93.5 ± 7.2 ^(a)^ 5.7	91.0 ± 12.7 ^(a)^ 7.2	92.8 ± 11.4 ^(a)^ 72.95 ± 2.32 ^(b)^ 6.8/5.9

**Table 3:** In vitro drug release studies for PLGA MSs (2–20 µm). Data represent the Dxm burst effect during the first 4 h (%), cumulative release (%), and content retained (μg/mg MSs) after 28 days of incubation at 37 °C in PBS and 0.1% sodium hyaluronate for Dxm ^(a)^ and FITC-HSA ^(b)^. Dxm release rate constants, K1 and K2, are also reported, all of which were obtained from data fitting the proposed biexponential release model (data are shown as $\bar{X}\pm \mathrm{SD}$, n = 6).

MS Formulation	Burst Effect (µg/mg MSs)	Dxm ^(a)^, HAS ^(b)^ % Released at 28 d	Dxm ^(a)^, HAS ^(b)^ Retained at 28 d (μg/mg MSs)	K_1_ (d^−1^)	K_2_ (d^−1^)	R^2^
F1 2–20 µm	6.63 ± 1.47	96.31 ± 2.76 ^(a)^	1.84 ± 0.26 ^(a)^	0.767 ± 0.02	0.351 ± 0.001	0.996
F2 2–20 µm	4.37 ± 1.66	88.96 ± 4.32 ^(a)^	3.65 ± 0.42 ^(a)^	0.704 ± 0.09	0.350 ± 0.002	0.988
F3 2–20 µm	2.45 ± 1.47	55.80 ± 1.31 ^(a)^ 40.19 ± 3.16 ^(b)^	10.20 ± 1.01 ^(a)^ 0.041 ± 0.002 ^(b)^	0.565 ± 0.03	0.354 ± 0.004	0.993

**Table 4:** Distribution extent (percentiles) for F1 MSs obtained when rpm and emulsification and/or maturation time are modified (data are shown as $\bar{X}\pm \mathrm{SD}$, n = 6).

Time	Emulsification	3 min		5 min	
	Maturation (PVA 0.1% *w*/*v*)	3 h	24 h	3 h	24 h
**Rpm**					
2500		d_10_ 34.96 d_50_ 82.21 d_90_ 121.13	d_10_ 43.38 d_50_ 99.18 d_90_ 136.28	d_10_ 28.53 d_50_ 74.29 d_90_ 116.30	d_10_ 32.91 d_50_ 82.93 d_90_ 128.19
5000		d_10_ 12.26 d_50_ 28.50 d_90_ 43.68	d_10_ 14.39 d_50_ 30.17 d_90_ 48.08	d_10_ 9.62 d_50_ 24.69 d_90_ 38.62	d_10_ 11.38 d_50_ 27.66 d_90_ 46.21
7500		d_10_ 7.56 d_50_ 17.35 d_90_ 34.43	d_10_ 9.13 d_50_ 21.39 d_90_ 38.43	d_10_ 5.87 d_50_ 14.19 d_90_ 31.53	d_10_ 7.16 d_50_ 18.21 d_90_ 36.38

## Data Availability

Data supporting reported results can be found in archived datasets in Universidad de Alcalá.

## Supplementary Materials

The following supporting information can be downloaded at: https://www.mdpi.com/article/10.3390/pharmaceutics18101239/s1. Figure S1: Dxm delivery data fitted to various kinetics models. Figure shows Higuchi and Peppas and Klose–Siepmann and Gallagher–Corringam models; Figure S2: MS’s formation mechanism. Figure shows how, in MSs at a high HSA loading level, more HSA is distributed near the surface area.
