# Supplementary material for: Optimization of a Dexamethasone and Vitamin E PLGA Microparticle Formulation Intended for Posterior Segment of the Eye to Cope with Patients’ Anti-Inflammatory Needs
Source: Pharmaceutics. 2026 Sep 29;18(10):1239. doi: 10.3390/pharmaceutics18101239 (PMC13708260; doi:10.3390/pharmaceutics18101239)
Supplement: Supplementary file 1 [file pharmaceutics-18-01239-s001.zip › pharmaceutics-4556940-supplementary.pdf]

Supplementary Materials

Optimization of a Dexamethasone and Vitamin E PLGA Microparticle Formulation Intended for Posterior Segment of the Eye to Cope with Patients’ Anti-Inflammatory Needs

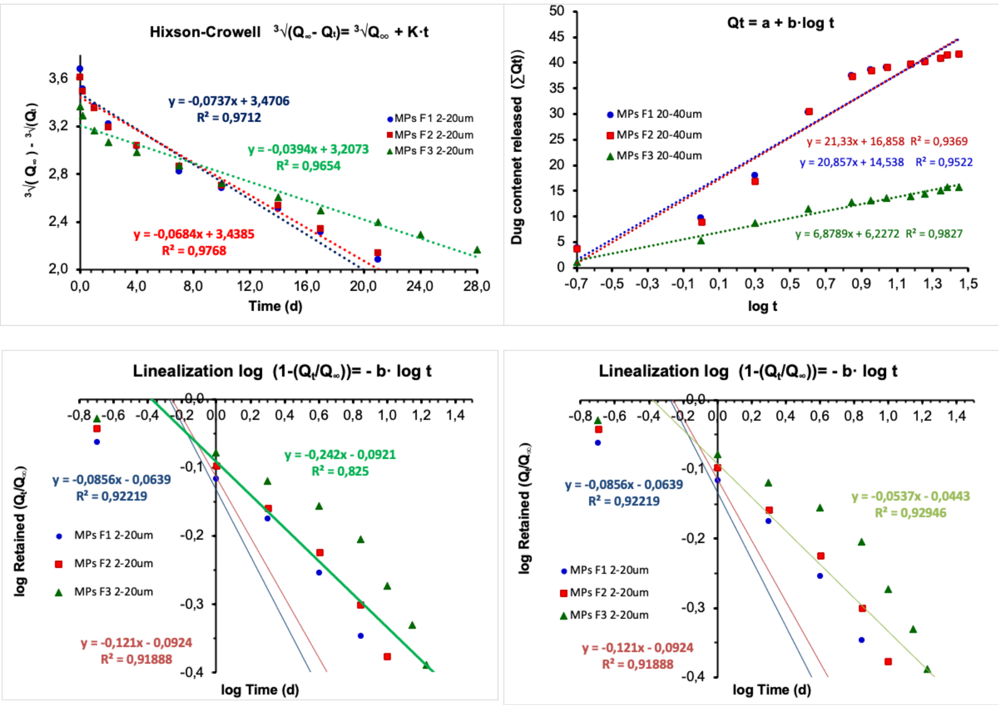

Figure S1: Dxm delivery data fitted to various kinetics models. Figure shows Higuchi and Peppas and Klose–Siepmann and Gallagher–Corringam models.

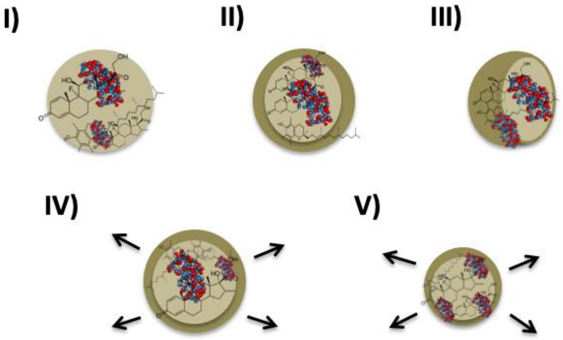

Figure S2: MS’s formation mechanism. Figure shows how, in MSs at a high HSA loading level, more HSA is distributed near the surface area.
